# Target delineation and optimal radiosurgical dose for pituitary tumors

**DOI:** 10.1186/s13014-016-0710-y

**Published:** 2016-10-11

**Authors:** Giuseppe Minniti, Mattia Falchetto Osti, Maximillian Niyazi

**Affiliations:** 1 Unit of Radiation Oncology, Sant’ Andrea Hospital, University Sapienza, Rome, Italy; 2 IRCCS Neuromed, Pozzilli (IS), Italy; 3 Department of Radiation Oncology, LMU Munich, Munich, Germany

**Keywords:** Fractionated stereotactic radiotherapy, Radiosurgery, Pituitary adenoma, Acromegaly, Cushing’s disease, Target delineation

## Abstract

Stereotactic radiosurgery (SRS) delivered as either single-fraction or multi-fraction SRS (2–5 fractions) is frequently employed in patients with residual or recurrent pituitary adenoma. The most common delivery systems used for SRS include the cobalt-60 system Gamma Knife, the CyberKnife (CK) robotic radiosurgery system, or a modified conventional radiotherapy machine (linear accelerator, LINAC). Tumor control and normalization of hormone hypersecretion have been reported in 75–100 % and 25–80 % of patients, respectively. Hypopituitarism is the most commonly reported late complication of radiation treatment, whereas other toxicities occur less frequently. We have provided an overview of the recent available literature on SRS in patients with a pituitary adenoma. Critical aspects of pituitary irradiation, including target delineation and doses to organs at risk, optimal radiation dose, as well as the long-term efficacy and toxicity of SRS for either nonfunctioning or secreting pituitary adenomas are discussed. Single-fraction SRS represents an effective treatment for patients with a pituitary adenoma; however, caution should be used for lesions > 2.5–3 cm in size and/or involving the anterior optic pathway. Future studies will be necessary to optimize target doses and critical organ dose constrains in order to reduce the long-term toxicity of treatments while maintaining high efficacy.

## Introduction

Conventional radiation therapy (CRT) has traditionally been used in patients with residual or recurrent secreting and nonfunctioning pituitary adenomas who have failed prior medical management and/or surgery, resulting in a variable long-term tumor control of 87–95 % at 10 years [1–4], and normalization of elevated plasma levels of growth hormone (GH) and adrenocorticotropic hormone (ACTH) in up to 55 %, and 78 % of patients, respectively [5–8]. Hypopituitarism occurs in 30–60 % of patients 5–10 years after irradiation, while other toxicities, including radiation-induced optic neuropathy, cerebrovascular accidents, and secondary tumors have been reported in 0–5 % [9–12].

Stereotactic radiosurgery (SRS) is a sophisticated radiation therapy technique that precisely delivers high dose of irradiation in a single o few (2–5) fractions to well-defined, small-to-moderate brain targets. SRS allows for more precise target localization and accurate dose delivery as compared with CRT, resulting in a reduction of the volume of normal brain tissue irradiated to high radiation doses [13]. The techniques used for the treatment of a pituitary adenoma involve the Gamma Knife (GK) [14], the CyberKnife (CK) robotic radiosurgery system [15, 16], or a modified conventional radiotherapy machine (linear accelerator, LINAC) [17, 18]. Data from literature report a tumor control after SRS up to 97 % at 5 years, with normalization of hormone hypersecretion in more than 50 % of patients [19]. Hypopituitarism is the most commonly reported late complication of treatment, whereas other late radiation-induced complications are low. As high doses are delivered to the tumor with the use of the stereotactic radiosurgical techniques, an accurate delineation of target and surrounding normal brain structures becomes increasingly important to minimize radiation-induced toxicity while maintaining high tumor control.

We aimed to provide a critical review of the different aspects of radiosurgical techniques for pituitary tumors, including the delineation of target and critical organs, technical characteristics of the different types of SRS delivery systems, the optimal dose and fractionation for nonfunctioning and secreting pituitary adenomas, and the long-term efficacy and toxicity.

## Methods and materials

A literature search was conducted in MEDLINE PubMed that evaluated adults with pituitary adenomas. The search focused on randomized, prospective and retrospective studies published in English. The searches were limited by date from January, 2000 to November, 2015 using a combination of medical subject headings (MeSH) (“pituitary adenomas/radiosurgery” or “nonfunctioning pituitary adenomas” or “acromegaly” or “Cushing disease” or “prolactinomas”) and free text terms (“toxicity” or “hypopituitarism” or “target delineation” or “radiosurgical dose” or “fractionated radiosurgery” or “organs at risk”). Articles were excluded from the review if they: had a non-English abstract, were not available through Pubmed, were pediatric series or case studies involving less than 8 patients, or were duplicated publications. To identify additional articles, the references of articles identified through the formal searches were scanned for additional sources. A total of 984 potentially relevant studies were identified. Finally, 92 studies reporting the clinical outcomes of SRS for either nonfunctioning or secreting pituitary adenomas with a minimum follow-up of 1 year were selected and included in the review.

## Target delineation

Defining the optimal target volume for a pituitary adenoma represents a balance between minimizing treatment-related toxicity while maintaining a high tumor control. Current optimal imaging technique for target delineation requires the use of precontrast and postcontrast magnetic resonance imaging (MRI) sequences to improve the accuracy of target identification and delineation. Contrast-enhanced 3D T1-weighted sequences with 1 mm thin slices are extremely useful for accurate target delineation by allowing identification of subtle enhancement patterns in the surrounding neurovascular structures and along the course of the optic nerve [20]. For planning purpose, MRI scan is subsequently fused with thin-slice non-contrast enahnaced CT scan. Although a displacement up to 2.8 mm has been reported for brain soft-tissue based fusion, the magnitude of displacement is considered negligible for lesions of the skull base due to its rigidity and great visibility in all imaging modalities [21]; so far, no additional margins would be required to ensure adequate target coverage during SRS to compensate fusion uncertainties. Since most pituitary adenomas are benign, slow-growing neoplasms, peritumoral edema is generally absent. For this reason, T2-weighted images, which are extremely useful in evaluating the parenchyma of the brain and the perilesional edema, are not generally used for target volume delineation. Preoperative MRI may be helpful to discern postoperative changes from tumor, especially in patients who had undergone several prior surgeries. Similarly, contrast-enhanced T1-weighted images with fat suppression may be used to minimize postoperative changes that might obscure the accuracy of radiosurgical targeting. when MRI is contraindicated, a thin-slice CT imaging through the pituitary regions is performed with and without contrast administration.

The gross tumor volume (GTV) is represented by the lesion visible on MRI/CT. The clinical target volume (CTV) includes microscopic disease. In general, additional margin expansion from GTV to CTV is unnecessary in pituitary adenomas; however, a small margin may be added in the intracavernous portion of aggressive adenomas to encompass potential areas of microscopic tumor infiltration. The planning tumor volume (PTV) should take into account uncertainties of patient setup. Currently, a similar sub-millimteric accuracy of target positioning has been reported for frameless CK and LINAC based systems (Novalis Tx) and frame based GK SRS technology [14–18, 22, 23]. In most centers, a margin of 0–1 mm is generally used for GTV to PTV expansion; however, due to the different commercial SRS systems, each department should audit their setup results and apply the margins on the basis of their own observations.

## Organs at risk

The sellar and parasellar region is an anatomically complex area including endocrine, nervous, and vascular structures. The pituitary fossa comprises the pituitary gland, which is composed of the adenohypophysis and neurohypophysis. The parasellar region encompasses the cavernous sinuses and the suprasellar cistern structures. The cavernous sinus consists of trabeculated, multilobulated venous channels which are located lateral to the sella turcica and sphenoid sinus. The cavernous sinus contains cranial nerves III (oculomotor), IV (trochlear), V1 (ophthalmic division of the trigeminal nerve), V2 (maxillary division of the trigeminal nerve) and VI (abducens). It also contains the cavernous segment of the internal carotid artery. The suprasellar cistern includes the optic chiasm and nerves, the anterior third ventricle, the hypothalamus, the pituitary infundibulum, the infundibular and suprachiasmatic recesses of the third ventricle.

**Fig. 1:**
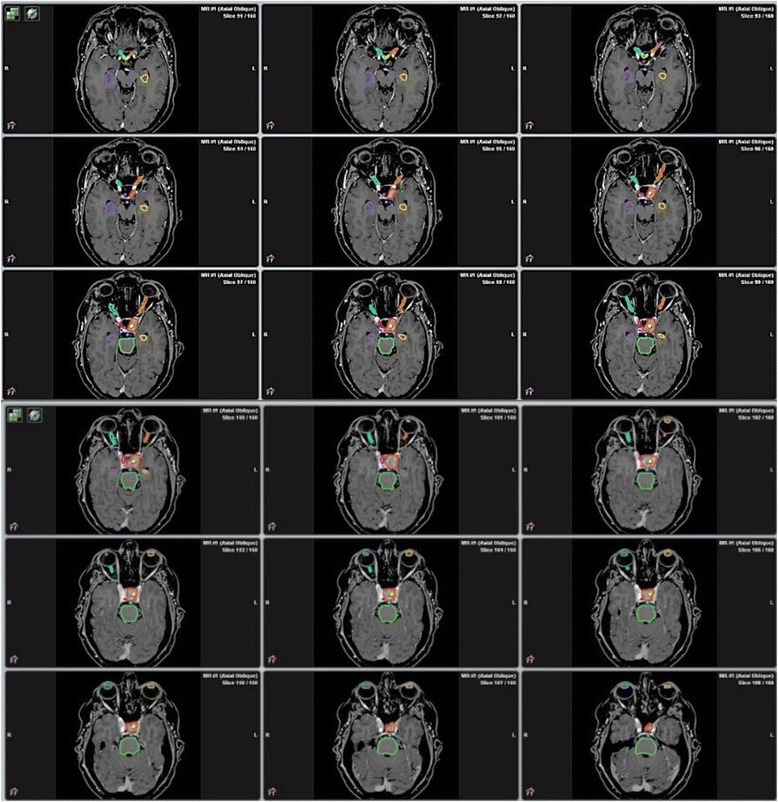
Target delineation of a pituitary adenoma. Gross tumor volume/Planning target volume (GTV/PTV) and organs at risk are outlined as solid lines. GTV/PTV (red); optic chiasm (yellow); left optic nerve (orange); right optic nerve (cyan); letf lens (light yellow); right lens (light blue); brainstem (green); pituitary stalk (blue); pituitary gland (pink); right hippocampus (purple); left hippocampus (golden yellow)

**Fig. 2:**
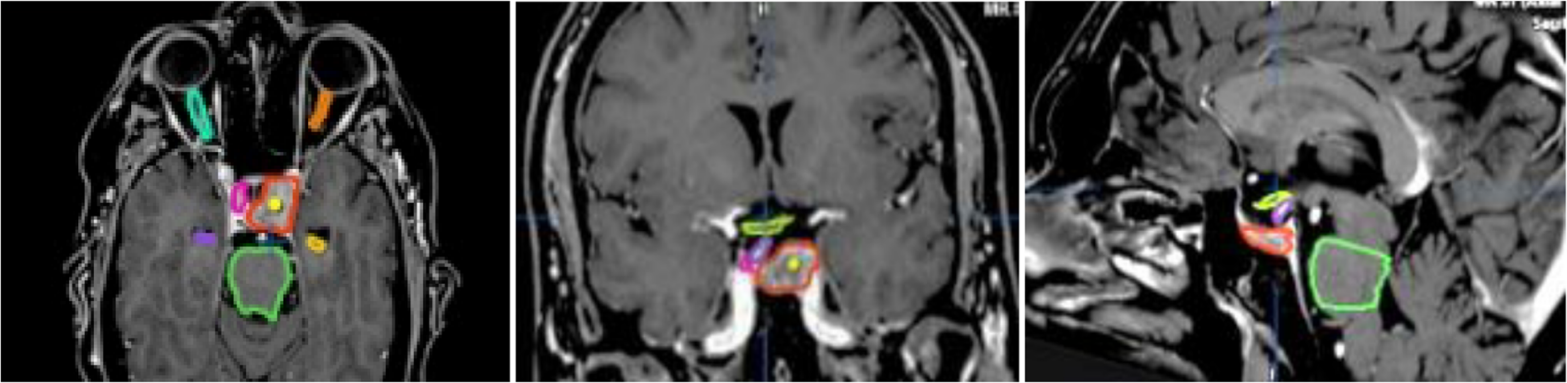
Axial, coronal, and sagittal view of target delineation for a pituitary adenoma. For GTV,PTV and organs at risk, see Fig. 1

A careful delineation of all organs at risk (OARs) surrounding the target volume is mandatory. OARs in the skull base region include optic nerves and chiasm, brainstem, pituitary stalk, pituitary gland, and cavernous sinus cranial nerves (an example of GTV and OARs contours is shown in Figs. 1 and 2). Expansion of OARs to create a planning risk volume (PRV) for each OAR may be applied; the margin, as for the GTV, should reflect the accuracy of daily set-up. Overlaps between PRVs and PTV should be considered; however, caution should be used when the reduction of the dose to the OARs may results in inadequate dose coverage of PTV. With regard to dose limits for the OARs, the optic nerves and chiasm are believed to be the most radiation-sensitive structures to SRS. A risk of radiation-induced optic neuropathy up to 2 % has been reported for point doses to the optic pathway of 8–10 Gy [24–31]; however, the risk of optic neuropathy remains low for point doses of 10–12 Gy to small portions of the optic apparatus [25, 27, 29, 30]. In a retrospective series of 222 patients who received GK SRS for benign tumors adjacent to the anterior visual pathway, Leavitt et al. [29] observed no new visual symptoms for patients receiving a maximum dose of 12 Gy to small portions (2–4 mm^3^) of the optic chiasm after single-fraction SRS. The risk of developing radiation-induced optic neuropathy was 0 for patients receiving a maximum point dose of 8–12 Gy and 10 % for those receiving a maximum point dose of 12–15 Gy to the anterior optic pathway. Hasegawa et al. [27] evaluated 100 patients undergoing GK SRS for craniopharyngiomas. Two patients who received maximum radiation point doses to the optic pathway of 15 and 18 Gy, respectively, developed optic neuropathy, whereas no visual deficits were observed in patients receiving lower doses. While these studies suggest that point doses up to 12 Gy to small portion of the optic pathway are associated with a low risk of optic neuropathy, in clinical practice a maximum point dose of 10 Gy is usually recommended when treating lesions adjacent to the optic pathway.

Little is known about the tolerance of the cranial nerves of the cavernous sinus. Leber et al. [25] reported no cranial nerve injury in patients receiving single-fraction SRS when doses of 5–30 Gy were delivered to the cavernous sinus. In contrast, Tishler et al. [24] reported a 13 % incidence of the third and sixth cranial nerve in 62 patients undergoing GK SRS; however, they could not find a significant relationship between the delivered dose of 10–40 Gy and new or worsening deficits. Although a precise tolerance dose of cranial nerves within the cavernous sinus after single-fraction SRS cannot be defined, doses up to 18 Gy to the cavernous sinus are associated with low incidence of radiation-induced toxicity (0–4 %) [13, 32].

Hypopituitarism is the most common adverse effect after SRS for a pituitary adenoma. Several studies have evaluated the relationship between radiation doses to the normal pituitary gland and distal infundibulum [33–38] and the development of hypopituitarism. Leenstra et al. [35] reported on 82 patients with either nonfunctioning or secreting pituitary adenomas who received GK SRS at the Mayo Clinic. Applying the criteria of a mean dose of 15 Gy to the pituitary gland, they noted new endocrine deficits in 12 of 40 patients (30 %) for doses < 15 Gy compared with 9 of 20 patients (45 %) who received a mean gland dose > 15 Gy. In their analysis they found new anterior deficits in 0 %, 29 %, 39 % and 83 % for mean doses to the pituitary gland ≤ 7.5Gy, 7.6–13.2 Gy, 13.3–19.1 Gy, and > 19.1 Gy, respectively. In another series of 85 patients treated with GK for a pituitary adenoma, Marek et al. [36] reported an incidence of hypopituitarism of 2.2 % for patients irradiated with a mean dose to pituitary < 15 Gy and 72.5 % for those who received a mean dose > 15 Gy. A significant correlation between the mean dose of 15 Gy to the pituitary gland and the development of new pituitary deficits has been reported in other studies [34, 38].

The correlation between the mean dose delivered to the pituitary stalk and the incidence of hypopituitarism has been evaluated in retrospective series [33, 34, 36, 38]. In a series of 130 patients treated with single-fraction SRS, Sicignano et al. [38] reported 5-year actuarial incidence of new pituitary deficits of 8 % for a mean dose to the pituitary stalk < 7.3 Gy and 32 % for a mean dose to the pituitary stalk > 7.3 Gy. Similarly, Feigl et al. [33] observed a significant incidence of new endocrine deficits for doses > 6.5 Gy to the pituitary stalk in a series of 108 patients treated with GK SRS for a pituitary adenoma. In contrast, Vladika et al. [34] found a significant incidence of new pituitary deficits after single-fraction SRS only for patients who received a maximum dose to the pituitary stalk > 17 Gy. Future prospective studies with an appropriate follow-up will be necessary to better identify the maximum safe doses to the pituitary gland and the pituitary stalk. Whenever possible, mean radiation doses to the pituitary gland and stalk should be kept under 12–15 Gy and 7–10 Gy, respectively, with the aim of limiting the development of new pituitary deficits.

Other OARs include the brainstem and hippocampi. For single fraction SRS, maximum brainstem doses of 12–14 Gy are associated with low (<5 %) risk of neurological complications, although this risk significantly increases for doses > 15 Gy given as single fraction [28, 39]. In a recent review of radiation associated brainstem toxicity, Mayo et al. [28] calculated a risk of normal tissue complication probability of 1 %, 13 %, 61 %, and 94 % for partial volume irradiation of one third of the brainstem to doses of 12.5, 14.2, 16, and 17.5 Gy, respectively. A lower risk of complications was observed when the same doses were delivered to a small partial volume (1 %) of the brainstem. Although definitive criteria of dose-volume effects on brainstem dose tolerance after single-fraction SRS remains to be better defined, in clinical practice caution should be used when delivering doses to the brainstem > 12.5 Gy. For tumors located in the parasellar region, hippocampi can be contoured as an effort to reduce the potential negative neurocognitive effect of high radiation doses to the hippocampal region [40]; the principle of this approach is acknowledged but there is currently insufficient evidence to support recommendations on hippocampal sparing during SRS.

There is limited evidence relating tolerance of the optic apparatus and cranial nerves of the cavernous sinus after multi-fraction SRS. Retrospective studies have observed a risk of optic complications of less than 1 % for patients with skull base tumors treated with doses of 21–25 Gy delivered in 3–5 fractions [41–46]. Liao et al. [45] reported the outcome of fractionated SRS delivered with a LINAC system. Thirty-four residual/recurrent pituitary adenomas with a median tumor volume of 4.11 cm^3^ in close proximity to the optic apparatus (median minimal distance 1 mm, ranging from 0 to 2.5 mm) were treated with a total dose fo 21 Gy in 3 fractions of 7 Gy each. With a median follow-up of 37 months, no patients developed optic neuropathy; the mean single-fraction doses to the optic nerve and chiasm were 5.58 ± 0.98 and 4.86 ± 0.15 Gy, respectively. One patients developed transient diplopia after SRS, which resolved after a short course of dexamethasone. Using doses of 21 Gy in 3 fractions or 25 Gy in 5 fractions delivered with CK, Iwata et al. [44] reported a grade 2 visual disorder in only 1 out of 100 patients at a median follow-up of 33 months; however, no details of doses delivered at optic apparatus were provided in their study. In another study of 34 patients who received a multifraction SRS (5 × 5 Gy) at University of Rome Sapienza for a skull base metastasis involving the anterior optic pathway, at a median follow-up of 13 months no optic neuropathy were observed for doses >25 Gy to less than one-third of optic chiasm and > 27.5 Gy to a small volume of 0.01–0.06 cm^3^ [46]. With regard to the cavernous sinus cranial nerves tolerance, no deficits have been reported using median doses of 20 Gy delivered in 2 to 5 fractions for perioptic lesions [41–43]. Although these studies indicate that 5 × 5 Gy or 3 × 7 Gy schedules are associated to a low risk of radiation-induced optic neuropathy and cavernous sinus cranial deficits, further studies need to better evaluate the dose-volume relations for OARs during multi-fraction SRS of patients with pituitary tumors.

## Treatment techniques

SRS for pituitary adenomas is typically delivered as single-fraction SRS or, less frequently, as multi-fraction SRS (2–5 fractions). Main used techniques include the use of GK, CK or a modified LINAC [13–18]. In its new version, GK uses 192 radioactive cobalt-60 sources that are spherically arrayed in a single internal collimation system via collimator helmets to focus their beams to a center point. The tungsten collimators are organized into eight sectors of 24 sources each with three different apertures of 4 mm, 8 mm, and 16 mm, respectively. A highly conformal but inhomogeneous dose distribution and high central tumor dose can be achieved through the optimal combinations of the number, the aperture and the position of the collimators [14, 15, 22]. Traditionally, patients are placed in a rigid stereotactic frame achieving submilimeter accuracy in dose delivery. The dose is typically prescribed at the 50 % isodose to obtain the maximum dose at the center of each pinpointed target and the prescribed dose at target edge.

CK (Accuray, Sunnyvale, CA) is a relatively new technological device that combines a mobile linear accelerator mounted on a robotic arm with an image-guided robotic system [15, 16, 23, 47]. Patients are fixed in a thermoplastic mask and the treatment can be delivered as single-fraction or multi-fraction SRS. A variable number of overlapping beams (up to 200) are delivered non-isocentrically to the target, resulting in excellent dose coverage to the target and conformity. The set of beam directions and analysis of dose distribution are chosen through an inverse planning process. During the treatment, acquired oblique digital X-ray images of the patients are compared with digitally reconstructed radiographs (DRRs), which are obtained from planning CT images, and positioning errors corrected by translating and rotating the treatment table with an accuracy of less than 1 mm [15, 16].

LINAC is the most frequently used device for delivery SRS in the world and uses multiple fixed fields or arcs shaped using a multileaf collimator with a leaf width of between 2.5 and 5 mm [17, 18, 48–51]. Dose conformity can be improved by the use of intensity modulation of the beams (IMRS) or volumetric modulated arc therapy (VMAT), resulting similar to that achieved with the GK and the CK. Patients are usually immobilized in a high precision frameless stereotactic mask fixation system with a reported accuracy of 1–2 mm [48]; however, technically most advanced LINACs offer improved accuracy of patient repositioning with the use of on-board imaging systems with either orthogonal x-rays or cone beam CT (CBCT) that achieves an accuracy of less than 0.5–1 mm [17, 18, 50, 51]. The ExacTrac®X.ray 6D system uses a combination of two main subsystems: an infrared-based system for initial patient setup and precise control of either translational or rotational couch movements, and a radiographic kV X-ray imaging system for position verification and readjustment based on internal anatomy. A CBCT system utilizes either the megavoltage radiation beam delivered from the LINAC or a kilovoltage beam delivered using an additional x-ray tube mounted on the LINAC. During a single 360° scan rotation, the system produces a series of two-dimensional images of the entire volume of interest from multiple projection angles, which can be reconstructed in a three-dimensional data that can be directly compared with the CT planning study.

The superiority in terms of dose delivery and distribution for each of these techniques remains matter of debate. Despite several differences in treatments-related parameters among GK, CK and LINAC, there are no comparative studies demonstrating the clinical superiority of a technique over the others in terms of local control and radiation-induced toxicity for patients with brain tumors. Regardless of the technology used, a robust quality assurance (QA) program, encompassing all clinical, technical, and patient-specific treatment aspects, is mandatory to ensure the accuracy and safety of cranial SRS [52]. As stated by The World Health Organization, proper QA measures are imperative to reduce the likelihood of accidents and errors and increase the probability that the errors will be recognized and rectified if they do occur [52]. For brain SRS, detailed equipment specifications and tolerances, as well procedures that minimize the risk of errors and incidents have been reported by several professional organizations [52–57].

## Clinical results

### Nonfunctioning pituitary adenomas

SRS is frequently used in patients with residual or recurrent nonfunctioning pituitary adenoma. Data for 1965 patients with a nonfunctioning pituitary adenoma included in 23 studies published between 2002 and 2015 are shown in Table 1 [32, 58–78]. SRS was performed with GK in 19 studies, LINAC in 3 studies, and CK in one study. With a median follow-up ranging from 21.7 months to 95 months (average 47.3 months), tumor control was seen in 94 % of patients using a median prescription dose of 16 Gy (range 12–20 Gy). In 9 studies including 1053 patients with nonfunctioning pituitary adenoma, 5-year Kaplan-Meier local control estimate was 92 % [59, 61, 64, 68, 72, 73, 76–78] (Table 1). A decrease in tumor size has been reported in 20–60 % of patients. With regard to factors predicting local control after SRS, smaller tumor volumes (<5 cm^3^) and limited suprasellar extension were associated with improved local control [68, 72, 73, 76].

**Table 1:** Selected published results of SRS (2000–2015) for the treatment of nonfunctioning pituitary adenomas

Authors	Patients	Type	dose	Follow-up	Tumor	Late toxicity (%)	
		of SRS	(Gy)	(months)	control (%)	visual	hypopituitarism
Feigl et al., 2002 [33]	61	GK	15^a^	55.2	94	NA	40
Sheehan et al., 2002 [58]	42	GK	16^a^	31.2	97.6	2.4	0
Wowra & Stummer, 2002 [59]	30	GK	16^a^	55	93.3 (93 at 5 years)	0	10
Petrovich et al., 2003 [60]	56	GK	15^a^	36	100	3	4
Losa et al., 2004 [61]	52	GK	16.6^a^	41	96.3 (88.2 at 5 years)	0	9.3
Muacevic et al., 2004 [62]	51	GK	16.5^a^	21.7	95	0	3.9
Picozzi et al., 2005 [63]	51	GK	16.5^a^	40.6	96.1	NA	NA
Iwai et al., 2005 [64]	34	GK	12.3^a^	59.8	87.1 (93 at 5 years)	0	6.5
Mingione et al., 2006 [65]	100	GK	18.5^a^	44.9	92.2	0	19.7
Voges et al., 2006 [66]	37	LINAC	13.4	56.6	100	1.4	12.3
Liscak et al., 2007 [67]	140	GK	20^a^	60	100	0	2
Pollock et al., 2008 [68]	62	GK	16^a^	64	96.8 (95 at 5 years)	0	27
Kobayashi et al., 2009 [69]	71	GK	14.1^a^	50.2	96.7	2.8	8.2
Hayashi et al., 2010 [70]	43	GK	18.2^a^	36	100	0	0
Gopalan et al., 2011 [71]	48	GK	18.4^a^	95	83.3	0	39
Iwata et al., 2011 [44]	100	CK	3×7/5×5	33	98	1	3
Park et al., 2011 [72]	125	GK	13^a^	62	90 (94 at 5 years)	0,8	24
Starke et al., 2012 [73]	140	GK	18^a^	50	89.6 (97 at 5 years)	0	30.3
Runge et al., 2012 [74]	61	LINAC	13	83	98	0	9.8
Wilson et al., 2012 [75]	51	LINAC	14	50	100	0	0
Sheehan et al., 2013 [76]	512	GK	16^a^	36	93.4 (95 at 5 years)	7.9	21
Lee et al., 2014 [77]	41	GK	12^a^	48	92.7 (85 at 10 years)	2.4	24.4
Bir et al., 2015 [78]	57	GK	15^a^	45.5	93 (90 % at 10 years)	0	8.8

*SRS* stereotactic radiosurgery, *GK* Gamma Knife, *LINAC* Linear Accelerator, *CK* CyberKnife, *NA* not assessed

^a^marginal dose

There is no consensus about the timing of SRS for nonfunctioning pituitary adenomas. Early postoperative SRS treatment has been suggested by some authors to decrease the rate of tumor progression and symptomatic endocrinophaty of subtotally resected nonfunctioning pituitary adenomas as compared with late SRS [63, 79]; in contrast, a policy of surveillance may be observed in older patients with small residual tumors for the low incidence of symtomatic recurrences following subtotal tumor resection [80].

New or worsened hormone pituitary deficits were the most common complication after SRS, with a median incidence of hypopituitarism of 18 % at median follow-up of 47 months [32, 58–78] (Table 1); neurological complications, including worsening of vision or other cranial nerve deficits, were less common (average 2.4 %, range 0–7.9 %).

Radiation doses used for patients with nonfunctioning adenomas treated with SRS are shown in Table 1. Median dose prescription was 12–14 Gy in 6 studies [64, 66, 72, 74, 75, 77], 14.1–16 Gy in 8 studies [32, 58, 59, 68, 69, 76, 78], and > 16 Gy in 7 studies [61–63, 65, 67, 70, 71, 73] including 349, 891, and 625 patients, respectively. Median tumor control rates were 93 % for doses of 12–14 Gy (median follow-up 61 months), 95 % for doses of 14.1–16 Gy (median follow-up 41 months), and 94 % for doses > 16 Gy (median follow-up 50 months). In a retrospective multicenter clinical trial of 512 patients treated with GK SRS, Sheehan et al [76] showed that margin doses < 12 Gy were significantly associated with worse control rate as compared with doses of 12–20 Gy, whereas no significant difference in tumor control rates have been observed between patients treated with 12–20 Gy versus those receiving doses > 20 Gy. Similar results have been reported by others [65, 71–73, 76].

Multi-fraction SRS (2–5 fractions) has been employed in patients with tumors involving the optic apparatus who are considered not suitable for SRS [44, 81–83]. Using doses of 18–24 Gy delivered in two to five sessions with Cyberknife, Adler et al. [81] reported a tumor control of 94 % in 46 patients with a pituitary adenoma or meningioma within 2 mm of the optic apparatus at a median follow-up of 49 months. A case of radiation optic neuropathy was observed in one patient who had a previous course of conventional RT. Iwata et al. [44] reported a local control rate of 98 % at 3 years in 100 patients with nonfunctioning pituitary adenomas treated with CK SRS using doses of 21 in 3 fractions or 25 Gy in 5 fractions. Complications were represented by grade 2 visual disorders in one patient and new onset of hypopituitarism in 4 patients. Similar tumor control and low toxicity have been reported in other few series [45, 82, 83].

### GH-secreting pituitary adenomas

SRS is commonly used in patients with a GH-secreting pituitary adenoma failing surgery and/or resistant to medical therapy. Data from 32 studies on SRS including 1802 patients with GH-secreting pituitary adenomas show median weighted tumor control and biochemical control of disease rates of 95 % and 44 %, respectively, at a median follow-up of 59 months (Table 2) [36, 37, 66, 70, 82, 84–110]. GK SRS is the most used technique, with a reported biochemical remission of 46 % at a median follow-up of 58 months. Four studies report results of LINAC SRS, 2 studies report results of proton SRS, and one study report results of CK SRS for GH-secreting tumors, showing a biochemical remission of disease ranging from 19 to 68 % at a median follow-up of 62 months.

**Table 2:** Selected published results of SRS (2000–2015) for the treatment of GH-secreting pituitary adenomas

Authors	Patients	Type	Dose	Follow-up	Tumor	Biochemical	Late toxicity (%)	
		of SRS	(Gy)	(months)	control (%)	remission (%)	visual	hypopituitarism
Zhang et al., 2000 [84]	68	GK	31^a^	34	100	40	NA	NA
Izawa et al., 2000 [85]	29	GK	22.5^a^	26.4	93	41	0	0
Attanasio et al., 2003 [86]	30	GK	20^a^	46	100	23	0	6.3
Jane et al., 2003 [87]	64	GK	15^a^	> 18	100	36	0	28
Castinetti et al., 2005 [88]	82	GK	28.5^a^	49.5	100	17	1.2	16
Gutt et al., 2005 [89]	44	GK	23^a^	23	100	48	0	NA
Kobayashi et al., 2005 [90]	67	GK	18.9^a^	63.3	100	17	11.1	14.6
Jezkova et al., 2006 [91]	96	GK	35^a^	53.7	100	50 (44 at 5 years)	0	27.1
Voges et al., 2006 [66]	64	LINAC	16.5	54.3	97	37.5 (33 at 5 years)	1,4	12.3 (18 at 5 years)
Petit et al., 2007 [92]	22	Protons	20	75	95	59	0	38
Pollock et al., 2007 [93]	46	GK	20^a^	63	100	50 (60 at 5 years)	2.2	36
Roberts et al., 2007 [82]	9	CK	18–24^a^	25.4	100	44.4	0	33
Vik-Mo et al., 2007 [94]	61	GK	26.5^a^	66	100	38 (58 at 5 years)	0	23
Jagannathan et al., 2008 [95]	95	GK	22^a^	57	98	53	4.2	34
Losa et al., 2008 [96]	83	GK	21.5^a^	69	97.6	60 (52 at 5 years)	0	8.5 (11.8 at 5 years)
Ronchi et al., 2009 [97]	35	GK	20^a^	114	100	82 (46 at 10 years)	0	50
Wan et al., 2009 [98]	103	GK	21.4^a^	67.3	95.1	36.9	NA	1.7
Hayashi et al., 2010 [70]	25	GK	25^a^	36	100	40	0	0
Iwai et al., 2010 [99]	26	GK	20^a^	84	96	38 (17 at 5 years)	0	8
Castinetti et al., 2009 [100]	43	GK	26^a^	96	100	42,0	0	23
Poon et al., 2010 [101]	40	GK	29^a^	73.8	NA	17	0	11.4
Erdur et al., 2011 [102]	22	GK	23.8^a^	60	95,2	54,5	0	28.6
Sheehan et al., 2011 [36]	130	GK	24^a^	31	93	53	2.3	34
Sicignano et al., 2012 [37]	39	GK	25^a^	60	97.7	54	NA	12.3
Franzin et al., 2012 [103]	103	GK	22.5^a^	71	97,3	60.7 (57 at 5 years)	0	7.8
Liu et al., 2012 [104]	40	GK	21^a^	72	97,5	47,5	0	40
Zeiler et al., 2013 [105]	21	GK	14.2^a^	33	100	30	3.9	13.2
Yan et al., 2013 [106]	22	LINAC	23	98	95	68.2	0	22.7
Wilson et al., 2013 [107]	86	LINAC	20	66	96	18.6	1,2	19.8
Lee et al., 2014 [108]	136	GK	25^a^	61.5	98.5	65.4 (73.4 at 6 years)	3.7	31.6
Wattson et al., 2014 [109]	50	Protons	20	51.5	100	48 (49 at 5 years)	0	57 (62 at 5 years)
Bostrom et al., 2015 [110]	21	LINAC	20	96	97.1	23	5	46.4

*SRS* stereotactic radiosurgery, *GK* Gamma Knife, *LINAC* Linear Accelerator, *CK* CyberKnife, *NA* not assessed

^a^marginal dose; ^1–3 fractions

The variable rate of hormone normalization observed in the different series may depend, at least in part, by different criteria used to define GH/IFG-1 plasma levels normalization, different follow-up times, pre-irradiation GH/IGF-1 levels and concomitant medical therapies, making difficult the interpretation of published results and the real efficacy of SRS. Nevertheless, using stringent criteria of cure, as defined by suppressed GH levels < 1 ng/ml during an oral glucose tolerance test (OGTT) and normal age-corrected IGF-1 levels, the Kaplan-Meier estimate of local control reported in 10 studies including 700 patients was 52 % at 5 years [66, 91, 93, 94, 96, 97, 99, 103, 108, 109] (Table 2), and normalization of GH/IGF-1 levels continued throughout the follow-up period.

A variable median dose prescription of 14 to 31 Gy has been used in the published series [36, 37, 66, 70, 82, 84–110] (Table 2). Median doses were < 20 Gy in 4 studies [66, 87, 90, 105], 20–25 Gy in 21 studies [85,8689,92,93,95–99,102–104,106–110], and > 25 Gy in 6 studies [84, 88, 91, 94, 100, 101] that include 216, 1196, and 390 patients, respectively (Table 2). Biochemical remission was 31 % for doses < 20 Gy (median follow-up 55 months), 47 % for doses of 20–25 Gy (median follow-up 60 months), and 33 % for doses > 25 Gy (median follow-up 59 months).

Although early reports suggest that the decline in GH levels after GK SRS is faster compared with fractionated RT [111, 112], the rate of decline observed in most recent series is similar to that reported following fractionated RT [86, 91, 93, 96, 100, 103]. The rate of decline mainly depends on pretreatment levels of GH and IGF-1 levels. Losa et al. [96] reported a median time for remission of 37 months for patients with pretreatment GH levels ≤ 7 μg/liter as compared with 93 months for patients with GH levels > 7 μg/liter. In another retrospective analysis of 46 patients, the 5-year biochemical remission rates 90 % for patients with IGF-1 levels less than 2.25 times the upper limit of normal and 38 % for those with IGF-1 levels greater than 2.25 times the upper limit of normal, respectively [93].

### Cushing disease

SRS data for 706 patients with Cushing’s disease included in 21 studies are shown in Table 3 [35, 37, 66, 69, 70, 92, 98, 105, 109, 113–123]. Biochemical remission of disease was reported from 25 % to 80.7 % of patients at a variable median follow-up of 2 to 17 years, with median tumor control rates ranging from 87 % to 100 %. At a weighted average follow-up of 56 months, the median tumor control was 95 % and biochemical remission of disease, as measured by normalization of 24 h urinary free cortisol (UFC) and/or plasma cortisol levels, was 48 %.

**Table 3:** Selected published results of SRS (2000–2015) for the treatment of ATCH-secreting pituitary adenomas

Authors	Patients	Type	dose	Follow-up	Tumor	Biochemical	Late toxicity (%)	
		of SRS	(Gy)	(months)	control (%)	remission (%)	visual	hypopituitarism
Izawa et al., 2000 [85]	12	GK	23.8^a^	26.4	100	17	NA	0
Sheehan et al., 2000 [113]	43	GK	20^a^	44	100	63	2	16
Hoybye et al., 2001 [114]	18	GK	>25^a^	17 years	100	83	0	66
Devin et al., 2004 [115]	35	LINAC	14.7	35	91	49	0	40
Voges et al., 2006 [66]	17	LINAC	16.4	58.7	82.4	52.9	1.4	12.3
Castinetti et al., 2007 [116]	40	GK	29.5^a^	54.7	100	42.5	2.5	15
Jagannathan et al., 2007 [117]	90	GK	25^a^	45	96	54	5.5	22
Petit et al., 2007 [92]	33	Protons	20	62	94	52	0	52
Pollock et al., 2008 [118]	8	GK	18^a^	54	100	87	0	36
Tinnel et al., 2008 [119]	12	GK	25^a^	37	83.3	50	0	50
Wan et al., 2009 [98]	68	GK	23^a^	67.3	89.7	27.9	2.9	1.7
Kobayashi et al., 2009 [120]	30	GK	28.7^a^	64.1	100	35	NA	NA
Hayashi et al., 2010 [70]	13	GK	25.2^a^	36	97	38	15.4	0
Sicignano et al., 2012 [37]	15	GK	23.8^a^	60	97.7	64	NA	12.3
Wein et al., 2012 [120]	17	LINAC	18	23	94.1	58.8	0	11.8
Zeiler et al., 2013 [105]	8	GK	24.7^a^	35	100	50	3.9	13.2
Grant et al., 2013 [121]	15	GK	35^a^	40.2	100	73	3.2	32
Sheehan et al., 2013 [122]	96	GK	22^a^	48	98	70	5	36
Wattson et al., 2014 [109]	74	Protons	20	47	98.6	67at 5 years	0	62 at 5 years
Wilson et al., 2014 [123]	36	LINAC	20	66	97	25	0	13.9
Marek et al., 2015 [35]	26	GK	29^a^	78	91.9	80.7	0	11.5

*SRS* stereotactic radiosurgery, *GK* Gamma Knife, *LINAC* Linear Accelerator, *CK* CyberKnife, *NA* not assessed\

^a^marginal dose

The median time to hormone normalization ranges from 12 to 25 months [35, 115, 116, 122]. In a retrospective series of 96 patients with Cushing’s disease treated by GK SRS at the University of Virginia, Sheehan et al. [122] reported a tumor control and biochemical remission rates of 98 % and 70 %, respectively, with a time to normalization of 16.6 months. New or worsened hypopituitarism occurred in 36 % of patients and progressive or new onset optic neuropathy occurred in 4.5 % of patients. In another series of 40 patients with Cushing’s disease treated by GK SRS, Castinetti et al. [116] reported the biochemical remission of disease in 42.5 % of patients at a mean follow-up of 54 months, with a mean time to hormone normalization of 22 months. Similar remission rates have been shown in other retrospective series [35, 115, 122] (Table 3). A recurrence rate up to 20 % after an initial remission of disease has been reported in some series [115, 116, 122, 123], indicating that a careful follow-up is mandatory also in patients who achieve normal hormone levels.

A median prescription dose of < 20 Gy has been used in 4 studies including 77 patients [66, 115, 118, 120], of 20 to 25 Gy in 11 studies including 487 patients [37, 85, 92, 98, 105, 109, 113, 117, 119, 122, 123], and > 25 Gy in 6 studies including 142 patients [35, 70, 114, 116, 121] (Table 3). The reported biochemical remission of disease was similar, being 53 % for doses < 20 Gy (median follow-up 40 months), 54 % for doses of 20–25 Gy (median follow-up 46 months), and 47 % for doses > 25 Gy (median follow-up 62 months), and with respective tumor control of 90 %, 98 %, and 95 %; however, in a few studies a higher margin radiation dose of 25 Gy was significantly associated with better biochemical remission of disease [117].

### Prolactinomas

SRS is usually reserved for prolactinomas resistant to medical therapy with dopamine agonists. Data for 610 patients with a prolactin-secreting pituitary adenoma included in 17 studies published between 2000 and 2015 are shown in Table 4 [32, 36, 60, 66, 85, 98, 100, 109, 118, 124–131]. SRS was performed with GK in 15 studies, with LINAC in one study, and with protons in one study. With a median follow-up ranging from 25 months to 75.5 months (average 49 months), tumor control and biochemical remission rates were reported for 95 % and 44 % of patients using median doses of 15 to 33 Gy.

**Table 4:** Selected published results of SRS (2000–2015) for the treatment of prolactin-secreting pituitary adenomas

Authors	Patients	Type	dose	Follow-up	Tumor	Biochemical	Late toxicity (%)	
		of SRS	(Gy)	(months)	control (%)	remission (%)	visual	hypopituitarism
Landolt 2000 [124]	20	GK	29	25	85	25	0	NA
Pan L et al., 2000 [125]	128	GK	33	41	99	41	0	NA
Izawa et al., 2000 [85]	15	GK	23.6	16	100	16	0	NA
Feigl et al., 2002 [32]	18	GK	15^a^	55	94	60	NA	40
Choi et al., 2003 [126]	21	GK	28.5^a^	42.5	96.9	23.8	0	0
Petrovich et al., 2003 [60]	12	GK	15^a^	41	83	83	0	4
Pouratian et al., 2006 [127]	23	GK	18.6^a^	55	89	26	7	28
Voges et al., 2006 [66]	13	LINAC	20	56	100	15.4	4.2	18.3
Pollock et al., 2008 [118]	11	GK	18^a^	48	100	18 at 4 years	9.1	36
Castinetti et al., 2009 [100]	15	GK	28^a^	96	100	46.6	0	21
Jezkova et al., 2009 [128]	35	GK	34^a^	75.5	97	37.1	0	14.3
Wan et al., 2009 [98]	176	GK	22.4^a^	67.5	90.3	23.3	0	1.8
Tanaka et al., 2010 [129]	22	GK	25^a^	60	100	18	4	42 at 4 years
Sheehan et al., 2011 [36]	32	GK	24^a^	31	93	26	2.4	24.4
Liu et al., 2013 [130]	22	GK	15^a^	36	86	27.3	0	4.5
Wattson et al., 2014 [109]	9	Protons	20	60	98	22	0	57
Cohen-Inbar et al., 2015 [131]	38	GK	25^a^	42.3	92	50	4.2	30.3

*SRS* stereotactic radiosurgery, *GK* Gamma Knife, *LINAC* Linear Accelerator, *CK* CyberKnife, *NA* not assessed

^a^marginal dose

The rate of normalization of prolactin levels was similar for patients treated with doses < 20 Gy (5 studies, 86 patients) [32, 60, 118, 127, 130], 20–25 Gy (7 studies, 305 patients) [36, 66, 85, 98, 109, 129, 131], and > 25 Gy (5 studies, 219 patients) [100, 124–126, 128] (Table 4). With median follow-ups of 50, 61, and 70 months, biochemical remission rates were 40 %, 23 %, and 38 % for doses < 20 Gy, 20–25 Gy, and > 25 Gy, respectively.

### Complications

Based on the available published series, the overall rate of serious complications after SRS is low. The mainly reported complication is the development of hypopituitarism, with 5-year incidence of new or worsening pituitary deficits of 24 % (range from 10 to 40 %) [34, 61, 64, 66, 68, 72–78, 91, 93–97, 99, 103, 108, 109, 116, 118, 127, 129]. Rates of hypopituitarism are similar among nonfunctioning and secreting pituitary adenomas. Factors related to higher risk of hypopituitarism include pre-existing anterior pituitary deficits, larger tumor volumes, higher doses delivered to the pituitary gland and to the pituitary stalk, and longer follow-up [34–37]. However, hypopituitarism can be effectively managed with hormonal replacement, and significant reduction of the prescribed dose to prevent hypopituitarism with the risk of compromising effectiveness of treatment in terms of local control and normalization of hormonal hypersecretion is not recommended. The risk of radiation-induced optic neuropathy is 0–3 % for single point doses less than 8–10 Gy to the optic apparatus [24–30]. Neuropathy of cranial nerves III–VI and radiation-induced brain necrosis have been reported in less than 2 % of patients, with higher risk for those who received previous conventional radiotherapy. The risk to develop a second brain tumor after SRS appears to be significantly less than that seen following conventional RT [11]; however the relatively short length of follow-up in several published series (< 5 years) does not allow for any definitive conclusion.

## Conclusions

SRS is an effective treatment modality for patients with pituitary adenomas after unsuccessful surgery and/or resistant to medical therapy. Doses of 13–16 Gy are usually employed for nonfunctioning pituitary adenomas with a reported tumor control of 85–95 % at 5–10 years, whereas higher doses are commonly used for hormonally active pituitary adenomas. For secreting adenomas, normalization of hormone hypersecretion is reported in more than 50 % of patients at 5 years, being similar for doses of 20–25 Gy or > 25 Gy. Currently, the optimal dose to achieve biochemical remission of hormone-secreting adenomas remains to be determined. The majority of studies report on the use of GK SRS in patients with either nonfunctioning or secreting pituitary adenomas, whereas only few retrospective series show the results of LINAC SRS. In the respect of few series, the reported tumor control, biochemical remission of disease, and toxicity so far are broadly equivalent.

Hypopituitarism represents the most commonly reported late complication of treatment, whereas the incidence of other late effect radiation complications are low. In this regard, an accurate delineation of the target and surrounding structures is mandatory during the radiosurgical process; future studies need to incorporate precise dosimetric information of doses delivered to OARs to better understand the relationship between doses to OARs and development of hypopituitarism.

A few series suggest that multi-fraction SRS may be an appropriate treatment in patients with tumors in close proximity to the optic apparatus; however, the advantages of hypofractionated schedules in terms of local control and risk of radiation-induced toxicity as compared to single-fraction SRS remains to be proved. For large pituitary adenomas involving the optic apparatus, the use of fractionated stereotactic radiotherapy using a conventional fractionation (45–54 Gy in 25–30 daily fractions) is recommended. Several studies have shown a tumor control of 90–95 % for pituitary tumors of any size, including large or giant tumors, and hormone hypersecretion normalization of 50 % at 5 years [132–142].

In clinical practice, single fraction SRS is recommended for small-to-moderate sized pituitary adenomas (< 2.5–3 cm) even when the adenoma is close to the optic apparatus as long as the dose to the optic apparatus is kept below 8–10 Gy. Fractionated SRS, usually 25 Gy in 5 fractions, may represent a better treatment option when a single fraction dose carries an unacceptable risk of optic neuropathy (as for tumors adiacent the optic chiasm); however, studies with more patients and longer follow-up are required to draw definite conclusions. Fractionated stereotactic radiotherapy would be the recommended radiation treatment modality for lesions > 3 cm in size and/or compressing the anterior visual pathway.
